# The prognostic role of palliative gastrectomy in advanced gastric cancer: a systematic review and meta-analysis

**DOI:** 10.1186/s12885-024-12860-z

**Published:** 2024-09-03

**Authors:** Desheng Luo, Hongtao Xu, Chuan Jiang, Jingjing Zheng, Dan Wu, Laizhen Tou, Haifeng Que, Zheng Sun

**Affiliations:** Department of Gastrointestinal Abdominal Hernia Surgery, Lishui Municipal Central Hospital, Lishui, 323000 Zhejiang China

**Keywords:** Prognosis, Advanced gastric cancer, Meta-analysis, Palliative gastrectomy

## Abstract

**Background:**

The effectiveness of palliative gastrectomy for advanced GC remains a topic of debate. This study sought to establish whether palliative gastrectomy has an impact on prolonging survival.

**Methods:**

We carried out systematic searches in PubMed, Cochrane Library, Web of Science, and the EMBASE databases from database inception to July 2023 to gather studies that examined the connection between palliative gastrectomy and the prognosis of advanced GC. The study employed overall survival as the primary outcome, with the hazard ratio serving as the selected parameter to gauge the association. Subgroup analyses were performed to delve into potential differences within the included studies, categorizing them by study region and sample size in order to examine possible sources of heterogeneity. The stability of individual studies was assessed through sensitivity analysis. The analysis included 20 articles, encompassing a total of 23,061 patients.

**Results:**

According to the meta-analysis results, patients who underwent palliative gastrectomy exhibited a noteworthy enhancement in overall survival (HR: 1.49; 95% CI: 1.12–1.99; *P* = 0.006) in comparison to those who did not receive this procedure. There was no association between the type of surgery and the length of hospital stay, as revealed by the analysis (HR = -0.02; 95% CI: -0.84–0.81; *P* = 0.970).

**Conclusions:**

Based on this meta-analysis, patients with advanced gastric cancer who underwent palliative gastrectomy may experience an extended survival duration without a significant prolongation of their hospitalization.

**Supplementary Information:**

The online version contains supplementary material available at 10.1186/s12885-024-12860-z.

## Background

Even with substantial advancements in diagnosis, experimental research, and therapeutic approaches, GC remains responsible for more than 6.8% of global cancer-related mortality and retains its position as the fifth key cause of cancer-related mortality, following female breast [1, 2]. Yet, recent years have seen significant progress in new treatment modalities and chemotherapy, leading to improved overall survival rates among GC patients with untreatable factors, as compared to those undergoing solely supportive treatment [3–5]. Timely diagnosis leads to improved long-term outcomes in cases of early GC, but when it comes to advanced GC with incurable factors, the outlook is less hopeful [6, 7]. Peritoneal dissemination, distant lymph node metastases, liver dissemination, lung metastases, and the presence of a significantly large primary tumor are the factors that classify patients with advanced gastric cancer as incurable [8]. Thus, palliative approaches remain essential for gastric cancer patients, particularly those in advanced stages [9].

In accordance with the guidelines from the National Comprehensive Cancer Network (NCCN), the use of gastric resections is advised primarily for symptom palliation, such as the management of uncontrollable bleeding orobstruction, in patients with incurable disease [10]. The guidelines established by the Japanese Gastric Cancer Association (JGCA) propose that patients diagnosed with metastases, yet not experiencing significant symptoms, may be eligible for treatment involving gastrectomy [11]. While surgical resection is generally deemed the most appropriate approach for treating GC, there remains ongoing debate concerning its application in cases of GC with incurable factors. Moreover, the guidelines established by the JGCA propose that patients diagnosed with oligometastatic disease may be eligible for weakly recommended surgical treatment following chemotherapy. Through palliative gastric resection, symptoms such as bleeding and obstruction can be alleviated and oral food intake can be restored [12, 13]. According to several research studies, gastric resection has been associated with potential advantages in terms of survival, symptom relief, and the enhancement of quality of life [13–17]. Conversely, a number of additional studies have noted that survival following palliative gastrectomy was linked to notable morbidity, extended hospitalizations, and diminished quality of life [18, 19]. It was recommended to consider gastrectomy only in cases characterized by severe complications, such as organ perforation or tumor bleeding [20, 21]. This study focuses specifically on palliative gastrectomy, which involves the surgical removal of part or all of the stomach for symptom relief in incurable cases, as opposed to other palliative surgeries that may not involve gastric resection.

Several review studies have explored palliative gastrectomy in patients with advanced, incurable GC. It is not difficult to find that the industry is increasingly clear about the therapeutic intent of palliative gastrectomy in patients with advanced, incurable GC and the positive impact on their survival outcomes. [22, 23] Given the timeliness of previous published studies and significant quality issues in some of the included trials, meta-analyses incorporating the latest findings are necessary to further identify the most appropriate surgical treatment strategies and patient populations. Therefore, we conducted this meta-analysis to assess the clinical relevance of palliative gastrectomy on overall survival for patients diagnosed with incurable advanced gastric cancer, paying particular attention to the criteria for patient selection and the choice of treatment strategy.

## Methods

This study is registered on the PROSPERO with the registration number: CRD42023454278.

### Systematic search strategy

A comprehensive and sensitive search strategy was created to include literature published from database inception to July 2023, ensuring a comprehensive coverage of relevant material. Electronic databases such as PubMed, Cochrane Library, Web of Science, and the EMBASE were employed in the comprehensive search process. Keywords such as “palliative gastrectomy,” “stomach neoplasm” and “gastric cancer,” were integrated into the search strategy. The search strategy was adapted for each database’s specific syntax and indexing terms while maintaining the core concepts of palliative gastrectomy and advanced gastric cancer. Two independent reviewers (Luo and Xu) were responsible for the article search process. In instances of discordance, they engaged in comprehensive discussions to achieve a consensus resolution. Titles and abstracts from studies considered potentially relevant were collected and incorporated into management software (EndNote^®^).

### Inclusion and exclusion criteria

Inclusion criteria: (1) Population: In this analysis, all the studies were comparative studies, specifically addressing patients with incurable advanced gastric cancer (GC). The core focus of these studies was the comparison between patients who received palliative gastrectomy and those who did not. In this context, advanced GC was explicitly defined using the TNM classification criteria, which encompassed T1–4N3M0, T4N1–3 M0, and any T or N with an M1 tumor designation [24, 25] The present study considered articles that included patients diagnosed with GC with metastasis, even if they did not utilize the TNM staging system. To ensure only incurable cases were included, we carefully reviewed the full texts of studies to confirm that patients had M1 or locally advanced disease deemed unresectable. Studies that did not clearly differentiate between potentially curable and incurable cases were excluded. (2) Intervention: effect of palliative surgery on prognosis in patients with advanced GC. We defined palliative gastrectomy as surgical removal of part or all of the stomach in patients with known incurable disease. Studies that did not clearly distinguish between curative-intent and palliative gastrectomies were excluded. (3) Outcome: overall survival, survival curves, median survival time and hazard ratio (HR), were included in the study to provide a comprehensive assessment of the data. (4) Study design: retrospective, cross-sectional, case-controlled, prospective studies, and randomized controlled trials (RCTs). Exclusion criteria: (1) In this study, only published studies that had undergone peer review and were available in journals were included. The exclusion criteria encompassed the exclusion of conference abstracts, reviews, protocols, letters, comments, articles lacking full-text, and any data that could not be obtained from the authors. (2) In instances where multiple investigations were conducted simultaneously by the same team, this study made the selection to include either the most recent publication or the article with the most extensive dataset. Additionally, any pertinent supplemental data were incorporated as needed.

### Data extraction and quality assessment of the included literature

Two researchers conducted the data collection and analysis, utilizing predefined tables with categories that encompassed authorship, publication date, geographical origin, study period, sample size, median survival duration, overall survival, and hospitalization duration. In instances where HR for overall survival were absent in the articles, the analysis utilized Engauge Digitizer 4.1 software to extract and compute HRs from the survival curves. Data extraction was undertaken by the first reviewer (Jiang), and the accuracy of the extracted data was verified through a thorough review performed by the second reviewer (Zheng).

The assessment of study quality in this meta-analysis was performed based on the Newcastle-Ottawa Quality Assessment Scale (NOS), ensuring rigorous evaluation of the included studies. For cohort studies, the NOS evaluated the quality of each study with regard to three domains: participant selection, comparability of groups, and the outcome of interest. The scoring system utilized in the NOS spans from 0 to 9, with studies that received scores surpassing 6 being deemed of high quality [26].

### Study outcomes

The primary outcomes were median survival in the two groups and the potential association of palliative gastrectomy for gastric cancer with overall survival. Secondary outcomes included length of hospitalization; post-operative complications and in-hospital mortality. Length of hospitalization analysis excluded patients with hospital mortality. Post-operative complications, as outlined in this study, comprised incidents that could be either surgically related or non-surgically related in origin. Hospital mortality, as defined in this study, encompassed deaths occurring either during the hospital stay or within 30 days following admission.

### Statistical analysis

The Tierney et al. method was utilized to calculate HRs and their corresponding 95%CIs based on the available data, in order to investigate potential associations with overall survival in the two study groups [27]. Length of hospitalization was compared based on the difference in mean and standard deviation of post-treatment length of stay between the control and intervention groups. The random-effect model was employed in this study to calculate the aggregated results for various study characteristics. The estimation of hospital stay for continuous outcomes involved the calculation of the standardized mean difference (SMD) along with a 95% confidence interval (CI). The overall effect size was determined using Cohen’s categories, which included the delineation of effect sizes into three categories: small (0.2–0.5), moderate (0.5–0.8), and large (> 0.8).

The assessment of statistical heterogeneity among the studies involved the use of Cochran’s Q test and the I^2^ statistic for quantitative evaluation, along with forest plots for visual examination. Heterogeneity was categorized as follows: 0–40%, considered not important; 30–60%, characterized as low heterogeneity; 50–90%, denoted moderate heterogeneity; and 75–100%, indicative of high heterogeneity. In cases where statistical heterogeneity was observed, subgroup analysis was performed to investigate the origin of heterogeneity. This analysis was based on two factors: the study area (China or non-China) and the sample size of studies (< 100 vs. ≥100). To gauge the possibility of publication bias, the research conducted visual screening of the Begg’s funnel plot and performed Begg’s test as an analytical tool to evaluate the presence or absence of publication bias. To enhance the reliability of our findings, a sensitivity analysis was carried out. The study implemented sensitivity analysis to assess the potential effect of individual studies on the overall results. This was accomplished by sequentially excluding one study at a time from the pooled analysis. The significance level for effect sizes in this research was defined at a *P* value of 0.05.

The statistical analysis in this study was carried out using Stata SE version 15.1 (Stata Corp, College Station, TX, USA). Reporting in this study was guided by the PRISMA Checklist.

## Results

### The included literature and methodological quality

**Fig. 1:**
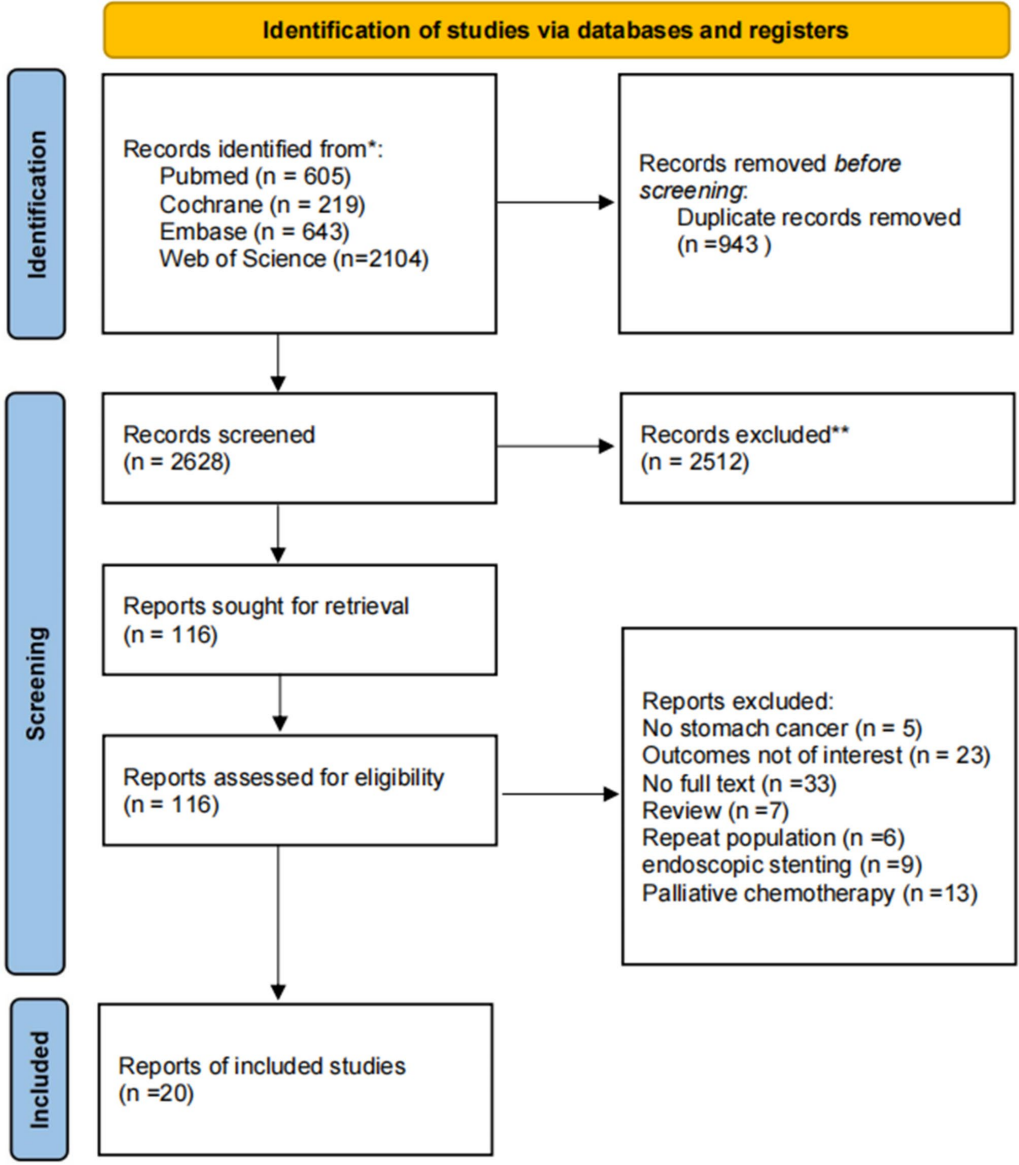
Flow diagram of study selection

From the initial literature search, a total of 3,571 articles were collected, with 605 originating from PubMed, 2,104 from Web of Science, 643 from Embase, and 219 from the Cochrane Library. After removing duplicate studies and filtering out non-relevant articles via the evaluation of article titles and abstracts, 116 studies that held promise for relevance underwent an in-depth review. In accordance with the pre-defined inclusion and exclusion criteria, this study encompassed a total of 20 studies, which collectively involved 23,061 patients diagnosed with advanced gastric cancer [15, 20, 28–46] Fig. 1 provides a visual representation of the literature search flow. The studies included in this analysis were published over a span of 18 years, from 2003 to 2021. All the articles considered in this study were observational trials, with a patient population of 5,431 individuals who underwent palliative gastrectomy and 17,630 individuals who did not undergo palliative surgery. The details regarding the characteristics of the studies and the results of the methodological quality assessment are presented in Table 1.

**Table 1:** Characteristic of the included studies

Study	Country	Time period	Intervention methods		Patients number (PG/NR)	Median survival time (PG/NR, month)	postoperative complication rates	In-hospital mortality	Quality scale
			Experimental	Control					
Cai 2003	China	1994–2001	PG	NR	64/31	11.3/6.4	NA	NA	6
Miner 2004	USA	1985–2001	PG	NR	147/160	8.3/13.5	49%/61%	7%/4%	6
Samarasam 2006	India	1999–2003	PG	NR	107/44	24.0/12.0	NA	NA	7
Saidi 2006	USA	1990–2000	PG	NR + Chem	24/81	13.2/5.5	33.3%/25%	8.3%/8.5	6
Huang 2010	China	1988–2008	PG	NR	365/151	10.2/4.5	NA	NA	6
Al-Amawi 2011	Poland	1998–2009	PG	NR	44/61	8.5/6.0	25%/11.5%	4.5%/4.8%	7
Chang 2012	Korea	1999–2004	PG	NR + Chem	162/95	4.8/4.1	NA	NA	6
Chen 2012	China	1993–2008	PG	NR	392/470	7.2/6.3	NA	NA	6
Yang 2015	China	2006–2013	PG	NR + Chem	114/153	14.0/8.6	14.91%/5.88%	NA	6
Nie 2016	China	2000–2014	PG	NR + Chem	183/188	11.9/9.3	10.4%/1.0%	NA	7
Chiu 2016	China	2008–2012	PG	NR + Chem	48/125	14.3/7.1	NA	NA	6
Yuan 2017	China	2000–2014	PG	NR + Chem	30/30	23.6/13.8	NA	NA	8
Hsu 2017	China	2000–2010	PG	NR	193/140	7.69/4.87	18.7%/20.0%	7.3%/4.3%	6
Omori 2019	Japan	2002–2014	PG	NR	40/19	4.8/2.9	25%/0%	10%/5.3%	6
Li 2019	USA	2004–2012	PG	NR	679/679	NA	NA	NA	7
Yang 2019	USA	2004–2013	PG	None	1006/6429	8.0/5.0	NA	NA	6
Peng 2020	USA	2010–2016	PG	NR + Chem	625/1250	12.0/6.0	NA	NA	7
Kamarajah 2021	USA	2010–2015	PG	None	1101/7417	12.8/1.8	NA	NA	6
Chen 2021	China	2000–2015	PG	NR	52/52	11.9/8.5	17.31%/19.23%	NA	7
Park 2021	Korea	2011–2017	PG	NR	55/55	28.4/7.6	NA	NA	8

### The primary outcomes

#### Median survival

**Fig. 2:**
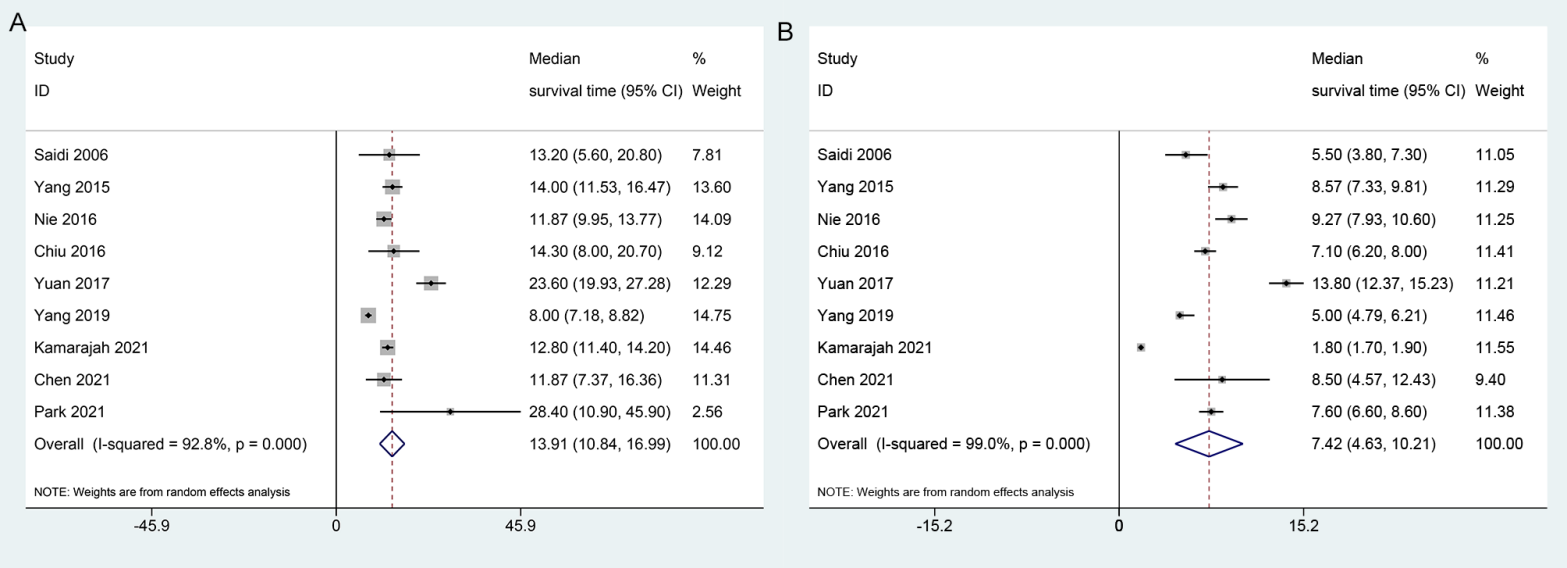
Forest plots show the association between palliative gastrectomy (**A**) non-gastrectomy (**B**) and overall survival

Nine of the included articles presented data related to median survival times. The calculated weighted average of the median survival time in the palliative gastrectomy group was 13.91 months (95% CI: 10.84–16.99), as illustrated in Fig. 2A; and within the non-gastrectomy group, the weighted mean of the median survival time was calculated to be 7.42 months (95% CI: 4.63–10.21), as depicted in Fig. 2B.

#### Overall survival

**Fig. 3:**
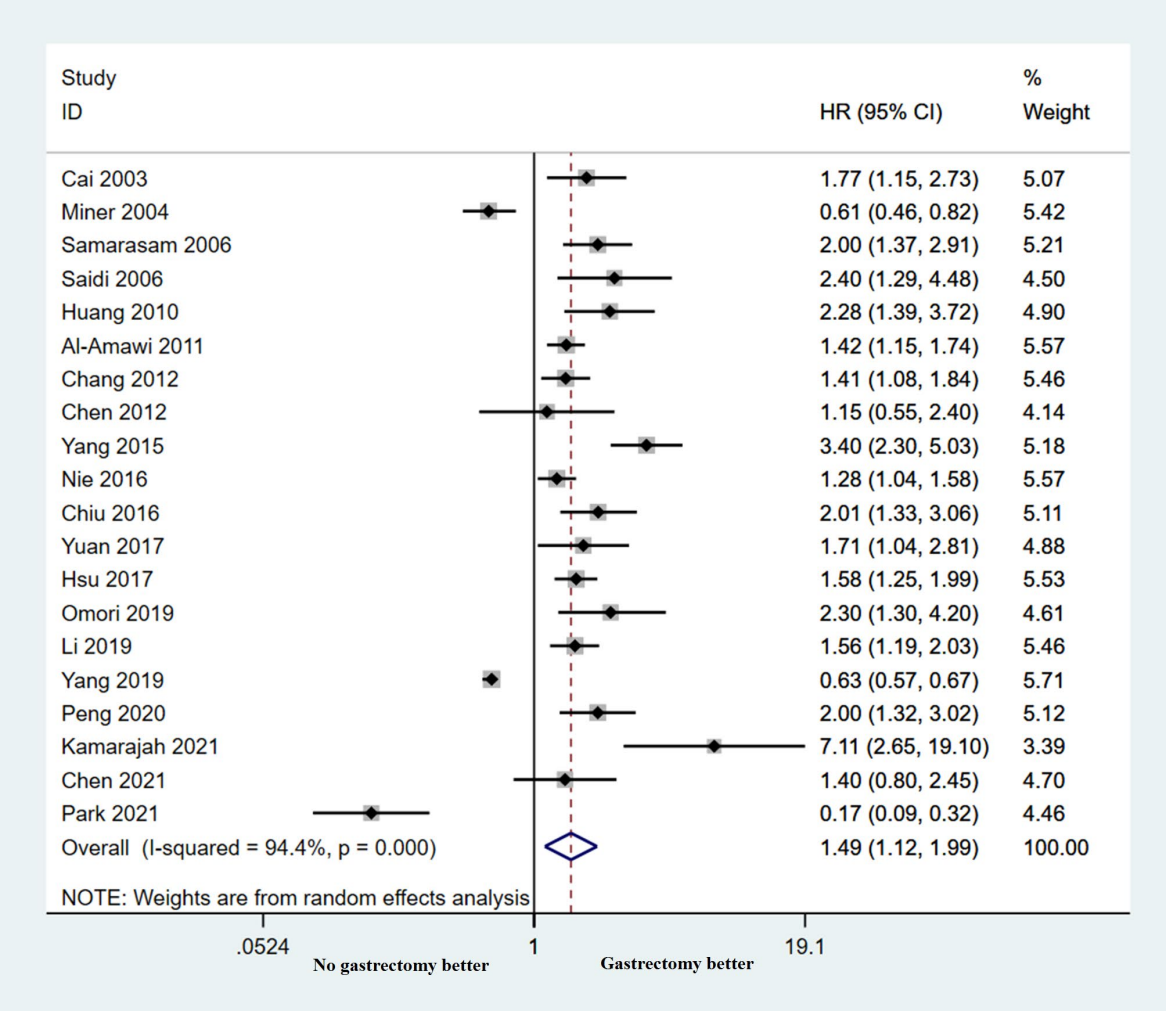
Forest plots show the association between palliative gastrectomy and overall survival outcomes

The 20 studies explored the association between palliative gastrectomy and OS outcomes. The prevailing results across the studies consistently pointed to the benefit of palliative gastrectomy in enhancing long-term survival for patients afflicted with incurable gastric cancer. The statistical significance of between-study heterogeneity was investigated, and the HR for overall survival was determined to be 1.49 (95% CI: 1.12–1.99; *p* = 0.006). The presence of significant heterogeneity (*P* < 0.001, I2 = 94.4%) was observed, and this is visually represented in Fig. 3.

### The secondary outcomes

#### Length of hospital stay

**Fig. 4:**
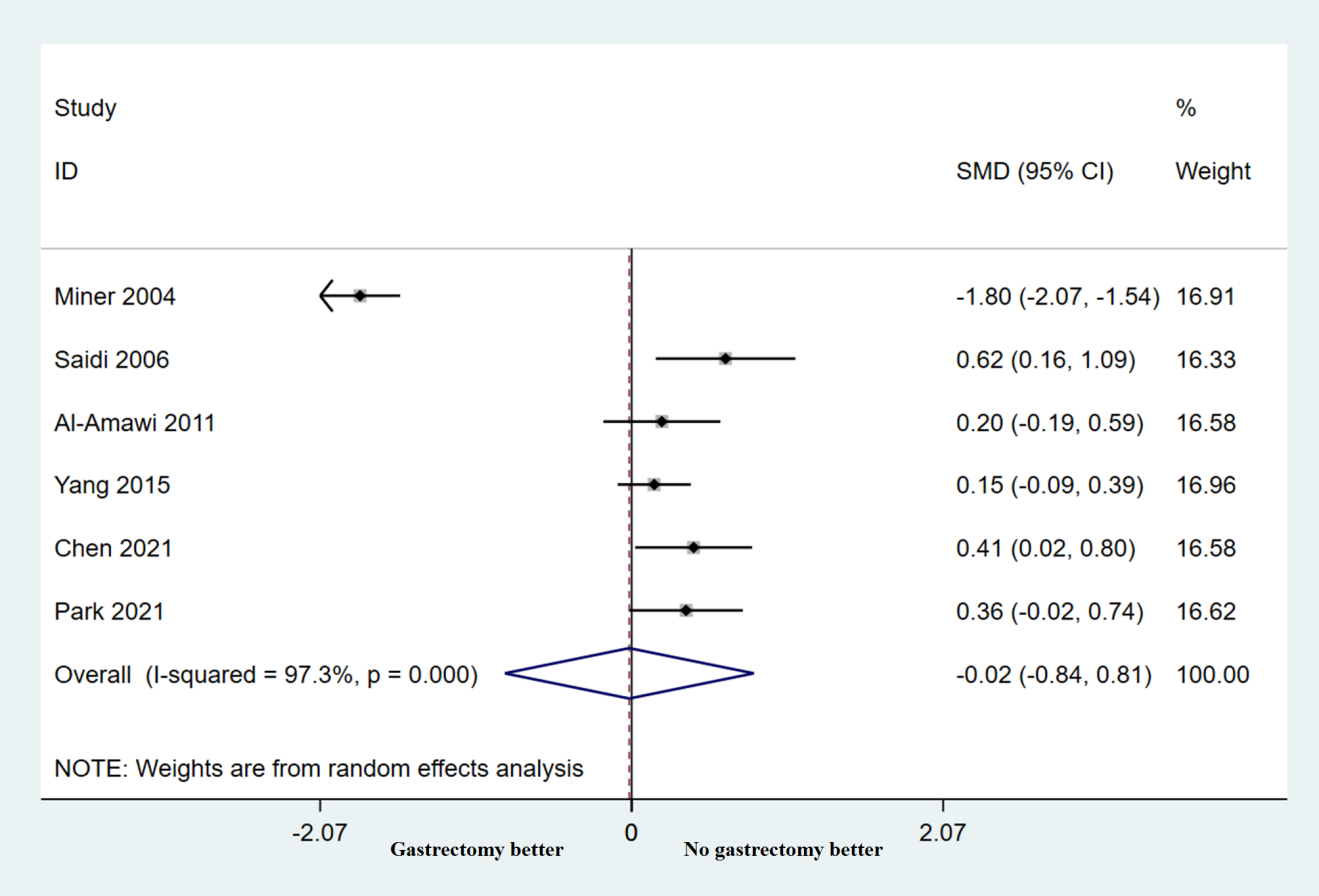
Forest plots show the length of hospital stay between palliative gastrectomy and non-resection procedure

A total of six studies participated in the length of hospital stay analysis of the relationship between palliative gastrectomy and non-resection procedure. The results showed that the length of hospital stay was not related to the type of surgery (HR =-0.02, 95% CI: -0.84–0.81, *P* = 0.970), and the heterogeneity across the studies was significant (*P* < 0.001, I2 = 97.3%; Fig. 4).

#### Post-operative complications

**Fig. 5:**
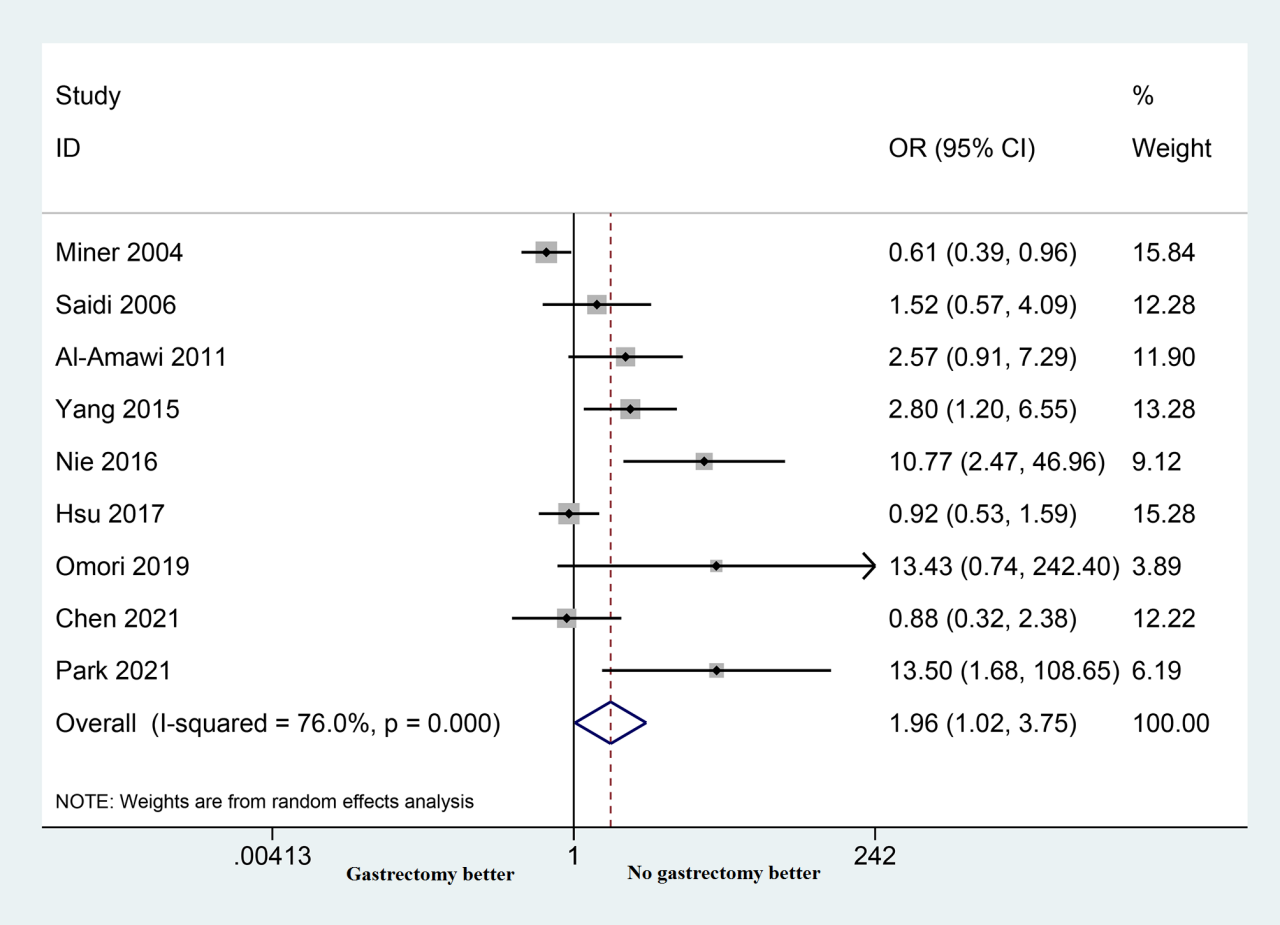
Forest plots show the post-operative complications in patients undergoing palliative gastrectomy and non-resection procedure

Nine studies reported data on the incidence of postoperative complications in patients undergoing palliative surgery and non-gastrectomy. Palliative gastrectomy was linked to a rise in overall complications when compared to non-gastrectomy surgery (OR = 1.96; 95% CI: 1.02–3.75; *p* = 0.042). Furthermore, there was significant heterogeneity observed across the studies (*P* < 0.001, I2 = 76.0%; as depicted in Fig. 5).

#### In-hospital mortality

**Fig. 6:**
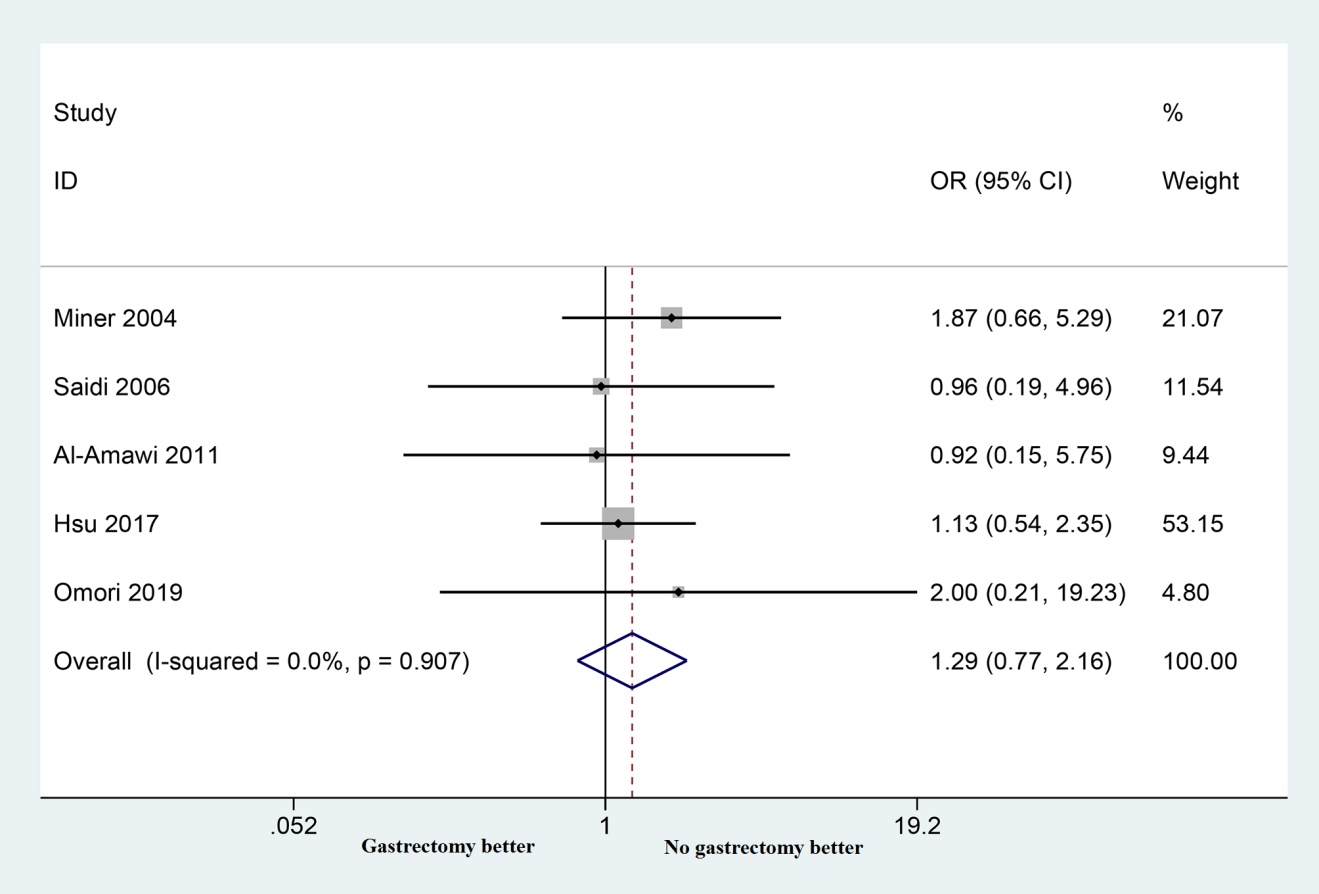
Forest plots show the in-hospital mortality comparing patients undergoing palliative surgery compared to non-gastrectomy

Data on in-hospital mortality, specifically comparing patients who underwent palliative surgery to those who did not, were reported in five studies. The analysis indicated that palliative surgery did not result in a statistically significant increase in in-hospital mortality when compared to non-gastrectomy surgery (OR = 1.29; 95% CI: 0.77–2.16; *p* = 0.337). Remarkably, there was no significant heterogeneity observed among the included studies (*P* = 0.907, I2 = 0.0%), as depicted in Fig. 6.

#### Meta regression analysis

Taking the effect size as the dependent variable, and year of publication, time period, country, number of people, and intervention method of the control group as the independent variables, Meta-regression analysis was conducted, and it was found that these variables did not significantly affect the effect size, and the specific results are shown in Table 2.

**Table 2:** Meta-regression for the association of palliative gastrectomy and the hazard ratio for overall survival

Variable	Standardized β coefficient	*P* value
Year of publication	0.405	0.471
Time period	0.418	0.422
Country	0.864	0.109
Number of people	0.923	0.292
Intervention method of the control group	0.319	0.619

#### Subgroup analysis

**Fig. 7:**
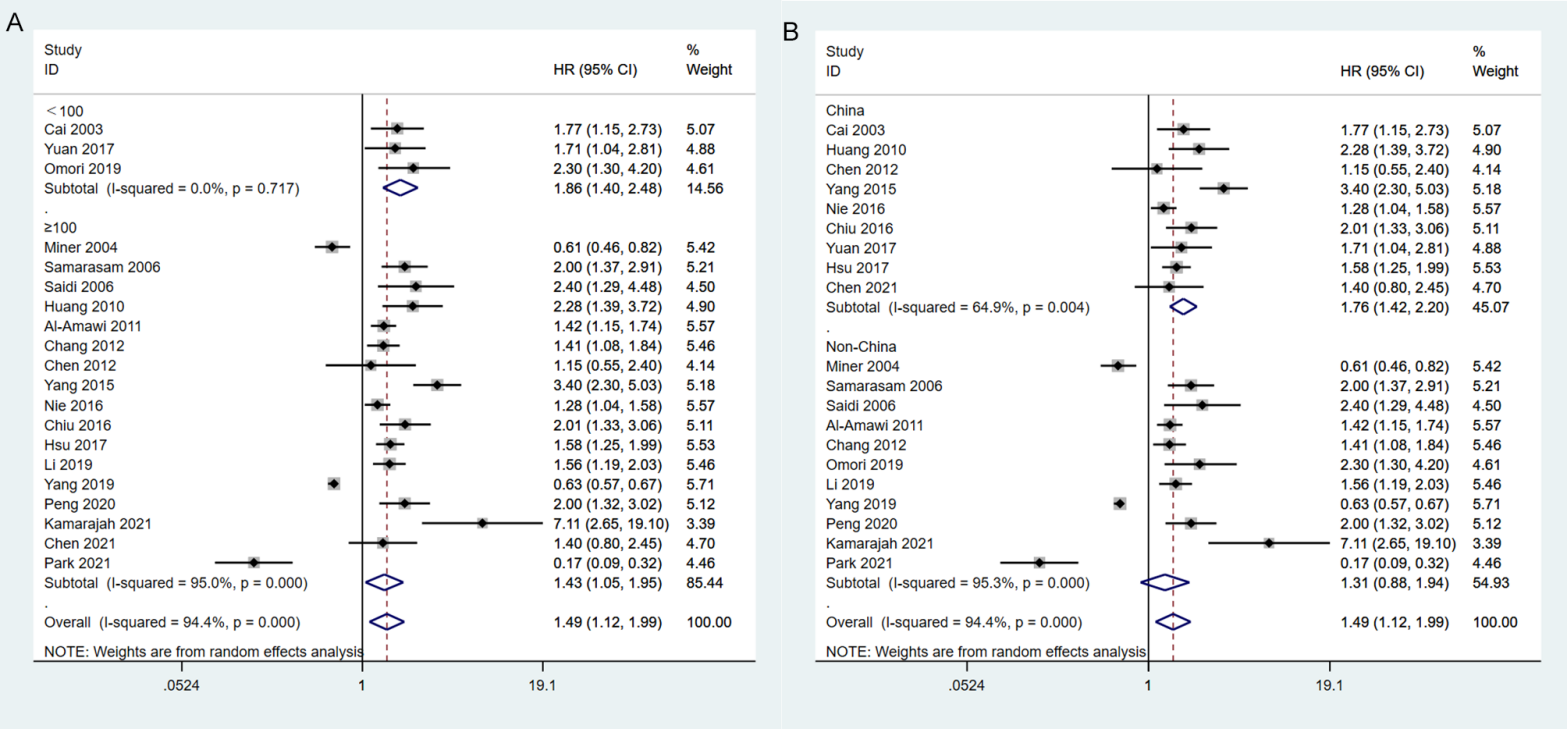
Forest plots show the association between palliative gastrectomy and overall survival stratified by the sample size (**A**) and studied area (**B**)

To investigate the likely causes of heterogeneity in the combined HR for overall survival, we carried out subgroup analyses by dividing eligible studies into subgroups based on study area (China vs. non-China) and sample size (< 100 vs. ≥100). The heterogeneity among the studies ceased to exist when they were categorized into two subgroups based on sample size. With regard to sample size, palliative gastrectomy was significantly correlated with longer OS (HR: 1.86; 95%CI:1.40–2.48; I2 = 0.0%; *P* < 0.001) in<100 compared with ≥ 100 (HR:1.49; 95%CI: 1.05–1.95; I2 = 95.0%; *P* = 0.022) (Fig. 7A). The subgroup analysis based on study area did not result in a significant modification of the prognostic impact of palliative gastrectomy on overall survival. However, it’s noteworthy that significant heterogeneity persisted across the studies, as shown in Fig. 7B.

#### Sensitivity analysis and publication bias

**Fig. 8:**
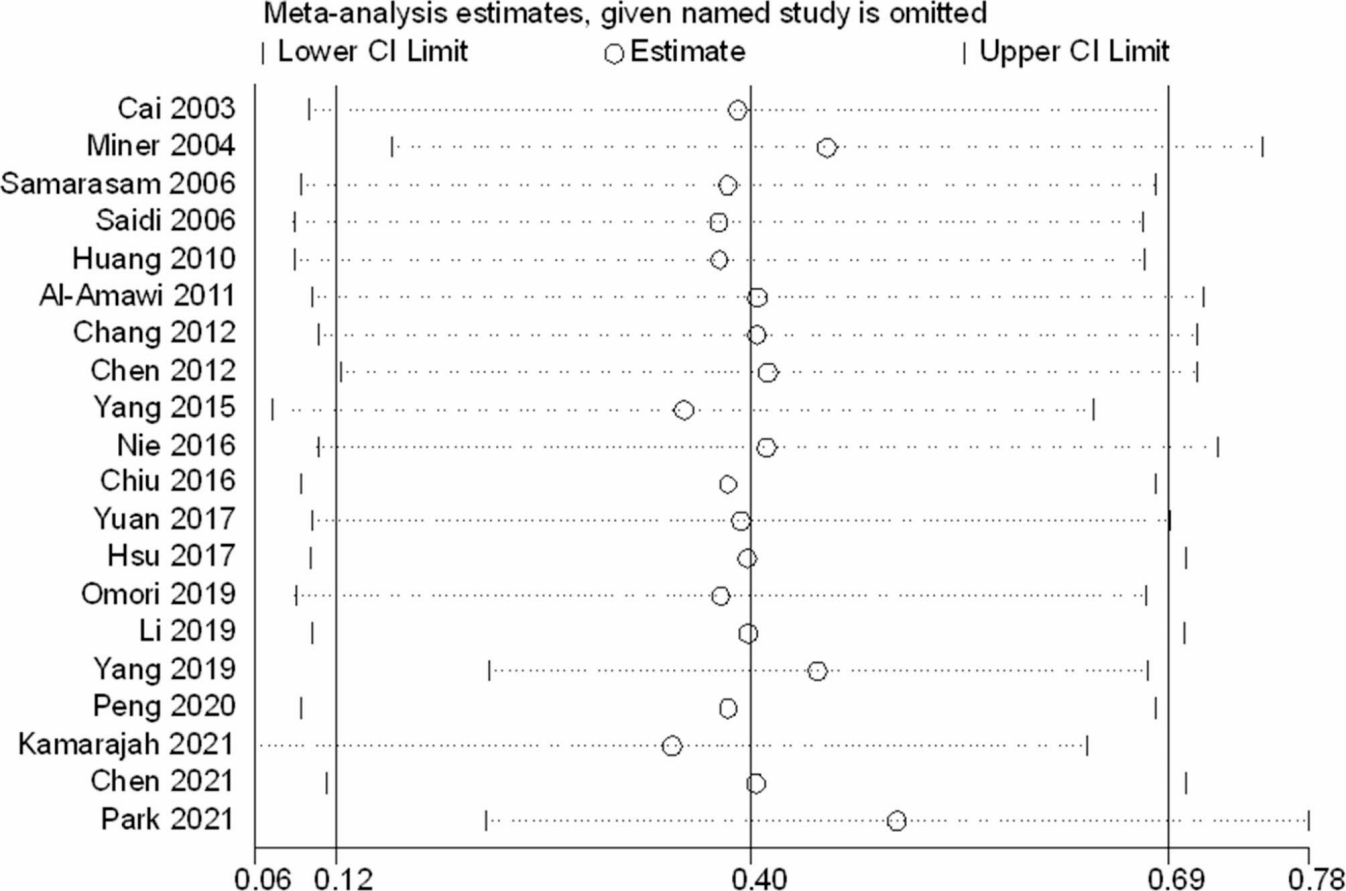
Sensitivity analysis examining the influence of individual studies on pooled results

**Fig. 9:**
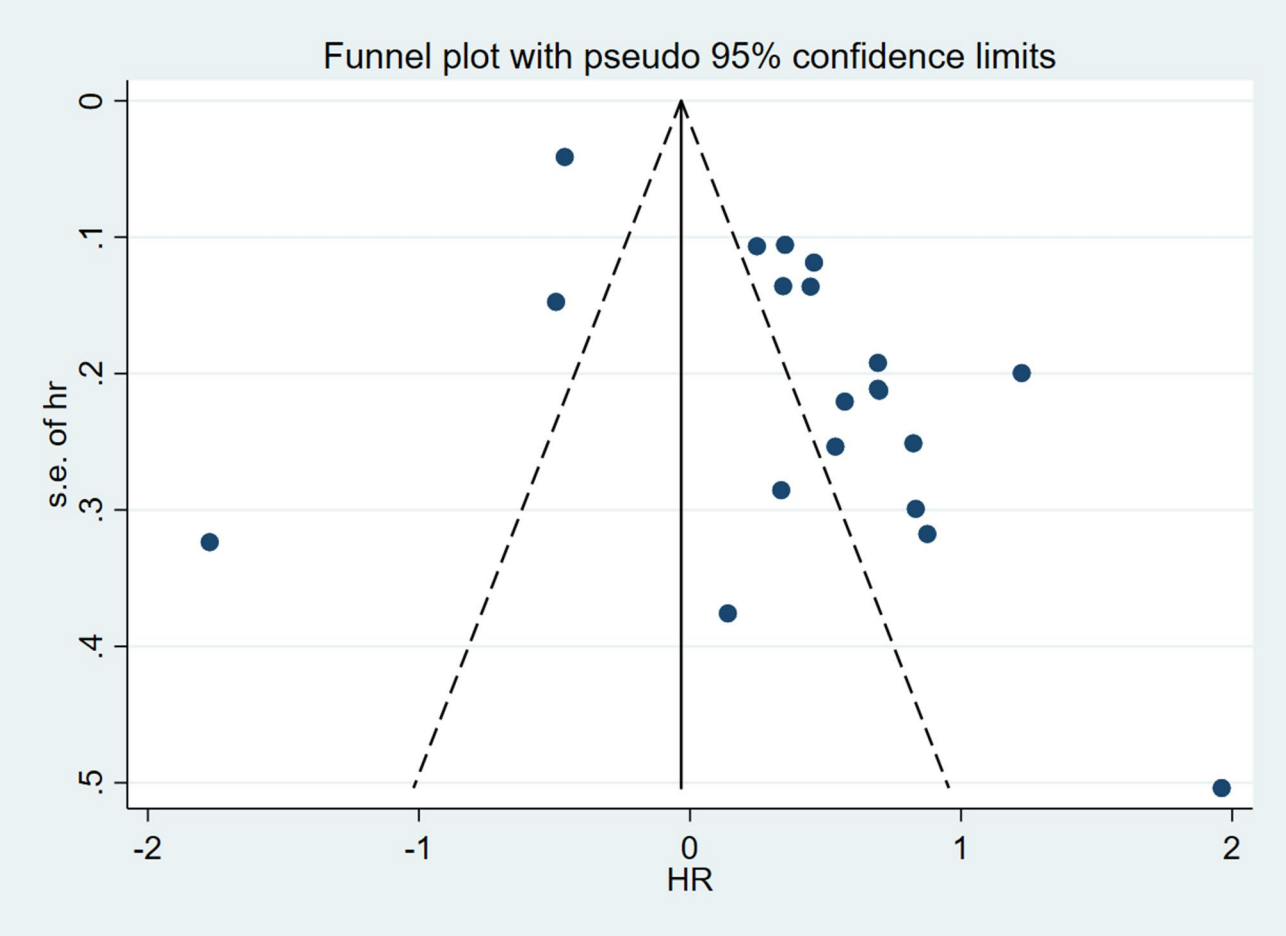
The Begg’s funnel plot for all included studies

Sensitivity analyses were then performed as part of the study. The sensitivity analyses involved the stepwise removal of a single study from the meta-analysis in order to reveal the influence of each individual dataset on the aggregated HR. The results from the sensitivity analyses conducted in this study demonstrated that the exclusion of any individual study had no notable effect on the meta-analytic outcomes. This underscores the robust nature of the results, as visually represented in Fig. 8. The results from the Begg’s funnel plot demonstrated considerable asymmetry for all the included studies, which is visually represented in Fig. 9. It’s noteworthy that, despite the visual asymmetry in the Begg’s funnel plot, the quantitative assessment using Begg’s test indicated the absence of statistically significant publication bias in the studies reporting overall survival (*P* = 0.347).

## Discussion

Over the course of the last 30 years, there have been transformations in the metastasis, recurrence, and survival characteristics of gastric cancer patients, accompanied by a decrease in the worldwide incidence of the disease. This trend has been accelerated by the implementation of strategies to combat Helicobacter pylori [47], advancements in standardized surgical techniques and supplementary tools, and enhancements in the overall quality of life across various societies, among other factors [48]. Nevertheless, patients diagnosed with GC are often already in an advanced stage. Recent advancements in chemotherapy protocols have led to enhanced survival rates among gastric cancer patients with incurable factors. Despite this, there is still a debate surrounding the suitability of palliative resection for patients diagnosed with advanced GC [49]. Hence, our study stands as the pioneering effort to conduct a meta-analysis on palliative resection in patients diagnosed with incurable advanced gastric cancer. The data revealed a discernible trend that implied palliative gastrectomy could be associated with enhanced survival in patients with advanced GC in an incurable state. Patients who undergo palliative gastrectomy experience hospital stays of similar duration to those who opt for non-resection procedures.

Almost all the articles in this study employed median survival time as the measure for evaluating the effect. Consequently, the overall survival rates derived from each article were considered suitable for examination within the scope of this study. In spite of notable heterogeneity, the results of this study still pointed towards an improvement in overall survival rates associated with palliative gastrectomy, reflecting an HR of 1.49 (95% CI 1.12–1.99).

The comprehensive meta-analysis by Cowling et al. provides important context for interpreting our results [50]. While they found improved 1-year survival with palliative gastrectomy, the benefits became less clear over time. This underscores the importance of considering both short-term and long-term outcomes when evaluating the role of palliative gastrectomy. Their observation of increased complications with palliative resections aligns with our findings of higher complication rates, emphasizing the need to carefully weigh potential benefits against risks. Three of other relevant systematic review and meta-analysis offers additional support for our findings [22, 23, 51]. Among these researches, Zheng et al.‘s observation that palliative gastrectomy plus chemotherapy improved overall survival compared to non-resection surgery plus chemotherapy is consistent with our results. However, their study also highlighted the potential benefits of specific surgical approaches, such as D1 + and D2 lymphadenectomy, which were not specifically addressed in our analysis. This suggests an area for future research to refine patient selection and surgical techniques for optimal outcomes. Morever, while affirming that palliative gastrectomy prolongs survival period, Sun et al. highlighted its superior performance in patients with stage M1 GCs [23].

While our results support the potential benefit of palliative gastrectomy, they also highlight the persistent challenges in this field. As noted by Lasithiotakis et al., the heterogeneity of patients with advanced gastric cancer makes it difficult to draw definitive conclusions about the universal applicability of palliative gastrectomy [52]. Our subgroup analyses, particularly the differences observed between studies from China and other regions, underscore the need for careful consideration of patient and tumor characteristics when deciding on treatment approaches.

The value of palliative gastrectomy for incurable patients depends on whether it improves survival and quality of life. In clinical practice, the benefits of surgery must be weighed against the risks and costs before a treatment decision is made. It’s important to note that the decision for palliative gastrectomy was typically made either upfront based on preoperative staging or intraoperatively upon discovering unresectable disease. The specific decision-making process varied among studies and was not always clearly reported. Quality of life is an important factor in assessing the effect of resection, but it is rarely mentioned in the articles included in the analysis. As we have limited data from retrospective trials and therefore cannot determine quality of life and costs, current evidence cannot elucidate potential clinical benefit or harm. However, Chang [30] used length of hospital stay as a parameter to evaluate quality of life. Our pooled results on length of hospital stay suggest that palliative gastrectomy may not reduce quality of life. Despite this, the question of whether palliative gastrectomy has an impact on quality of life continues to be a matter of contention, highlighting the requirement for future research.

In multiple Asian nations, early diagnosis and prevention strategies are rigorously executed [53], yielding a greater proportion of early-stage tumor diagnoses and improved prognostic outcomes for individuals with gastric cancer when compared to their European and Western counterparts. Discrepancies in the outcomes could be attributed to variances in geographical regions. Among the articles included in this study, nine were contributed by China, while the remaining eleven were from sources outside of China. Subgroup analyses were performed by region to investigate whether regional differences had an influence on the analysis. According to our results, the potential benefit of palliative gastrectomy on survival was more prominent in China, while the dataset exhibited noticeable heterogeneity. Before arriving at a definitive conclusion, it is essential to gather additional data from non-Chinese sources for comparison with the existing Chinese data.

It’s important to note that there are certain limitations in our study. First, while our meta-analysis included studies from various institutions, including large database studies like those using SEER data, it’s important to note that many of the included studies were single-institution retrospective analyses. This mix of study types contributes to the heterogeneity observed in our results. It’s important to note that the second limitation of our study lies in the fact that the decision to opt for either palliative gastrectomy or non-resection procedures was based on a case-by-case assessment conducted by the surgical team. The third limitation is related to the fact that we did not have access to data on performance status, quality of life metrics, or detailed information about the chemotherapy regimen. The observed heterogeneity in the included studies was largely attributed to disparities in study design as well as variations in treatment modalities, such as single-agent versus combination chemotherapy regimens and chemotherapy with or without non-resection surgery. Fourth, our analysis of hospital stay length has limitations. Patients who received outpatient treatments like chemotherapy were not captured in this metric. This could potentially skew the results and should be considered when interpreting the findings. Finally, future research endeavors may find it worthwhile to explore the possibility of surgical resection following chemotherapy as a strategy to enhance the survival of patients with advanced GC. Substantial emphasis should be placed on conducting high-quality multicenter clinical RCTs in the future to meticulously examine the influence of gastrectomy on overall survival (OS).

Finally, it should be acknowledged that this study does not encompass data on long-term complications, morbidity, and the quality of life of patients in the aftermath of gastrectomy. It is vital to recognize that, for a subset of patients, these considerations play a pivotal role in their treatment decisions. Hence, future studies on this topic should prioritize a more robust investigation of these elements.

## Conclusions

The results of this meta-analysis demonstrate that palliative gastrectomy confers a statistically significant survival advantage for individuals diagnosed with incurable advanced GC, while not imposing a substantial increase in the duration of hospitalization. However, further validation of the survival advantages linked to palliative gastrectomy in advanced GC patients necessitates the execution of prospective trials and RCTs.

## Electronic supplementary material

Below is the link to the electronic supplementary material.

Supplementary Material 1

## Data Availability

All data generated or analysed during this study are included in this article.

## Abbreviations

NCCN: National comprehensive cancer network
JGCA: Japanese gastric cancer association
RCTs: Randomized controlled trials
NOS: Newcastle-Ottawa quality assessment scale
SMD: Standardized mean difference
CI: Confidence interval

## References

[1] Shibata A, Parsonnet J. Stomach cancer. In Cancer Epidemiology and Prevention. 3rd edition. Schottenfeld D, Fraumeni J, editors. New York: Oxford University Press; 2006.

[2] Parkin P D M, Bray F, Ferlay J, Pisani P. Global cancer statistics, 2002. Cancer J Clin. 2005;55:74–108. 10.3322/canjclin.55.2.74 15761078

[3] Koizumi W, Narahara H, Hara T, Takagane A, Akiya T, Takagi M, et al. S-1 plus cisplatin versus S-1 alone for first-line treatment of advanced gastric cancer (SPIRITS trial): a phase III trial. Lancet Oncol. 2008;9:215–21. 18282805 10.1016/S1470-2045(08)70035-4

[4] Glimelius B, Ekström K, Hoffman K, Graf W, Sjödén PO, Haglund U, et al. Randomized comparison between chemotherapy plus best supportive care with best supportive care in advanced gastric cancer. Annals Oncology: Official J Eur Soc Med Oncol. 1997;8:163–8. 10.1023/A:1008243606668 9093725

[5] Pyrhönen S, Kuitunen T, Nyandoto P, Kouri M. Randomised comparison of fluorouracil, epidoxorubicin and methotrexate (FEMTX) plus supportive care with supportive care alone in patients with non-resectable gastric cancer. Br J Cancer. 1995;71:587–91. 7533517 10.1038/bjc.1995.114 PMC2033628

[6] Kakeji Y, Maehara Y, Tomoda M, Kabashima A, Ohmori M, Oda S, et al. Long-term survival of patients with stage IV gastric carcinoma. Cancer. 1998;82:2307–11. 9635521 10.1002/(SICI)1097-0142(19980615)82:12<2307::AID-CNCR2>3.0.CO;2-P

[7] Isobe Y, Nashimoto A, Akazawa K, Oda I, Hayashi K, Miyashiro I, et al. Gastric cancer treatment in Japan: 2008 annual report of the JGCA nationwide registry. Gastric cancer: Official J Int Gastric Cancer Association Japanese Gastric Cancer Association. 2011;14:301–16. 10.1007/s10120-011-0085-6 PMC3196643 21894577

[8] Maehara Y, Hasuda S, Koga T, Tokunaga E, Kakeji Y, Sugimachi K. Postoperative outcome and sites of recurrence in patients following curative resection of gastric cancer. Br J Surg. 2000;87:353–7. 10718807 10.1046/j.1365-2168.2000.01358.x

[9] Miner TJ, Karpeh MS. Gastrectomy for gastric cancer: defining critical elements of patient selection and outcome assessment. Surg Oncol Clin N Am. 2004; 13:455 – 66, viii. 10.1016/j.soc.2004.03.004 15236728

[10] Ajani JA, Bentrem DJ, Besh S, D’Amico TA, Das P, Denlinger C, et al. Gastric cancer, version 2.2013: featured updates to the NCCN guidelines. J Natl Compr Cancer Network: JNCCN. 2013;11:531–46. 23667204 10.6004/jnccn.2013.0070

[11] Japanese Gastric Cancer A. Japanese classification of gastric carcinoma – 2nd English Edition. Gastric cancer: Official J Int Gastric Cancer Association Japanese Gastric Cancer Association. 1998;1:10–24. 10.1007/PL00011681 11957040

[12] Monson JR, Donohue JH, McIlrath DC, Farnell MB, Ilstrup DM. Total gastrectomy for advanced cancer. A worthwhile palliative procedure. Cancer. 1991;68:1863–8. 1717128 10.1002/1097-0142(19911101)68:9<1863::AID-CNCR2820680902>3.0.CO;2-1

[13] Haugstvedt T, Viste A, Eide GE, Söreide O. The survival benefit of resection in patients with advanced stomach cancer: the Norwegian multicenter experience. Nor Stomach Cancer Trial World J Surg. 1989; 13:617 – 21; discussion 21 – 2. 10.1007/BF01658884 2479177

[14] Medina-Franco H, Contreras-Saldívar A, Ramos-De La Medina A, Palacios-Sanchez P, Cortés-González R, Ugarte JA. Surgery for stage IV gastric cancer. Am J Surg. 2004;187:543–6. 15041508 10.1016/j.amjsurg.2003.12.045

[15] Samarasam I, Chandran BS, Sitaram V, Perakath B, Nair A, Mathew G. Palliative gastrectomy in advanced gastric cancer: is it worthwhile? ANZ J Surg. 2006;76:60–3. 16483298 10.1111/j.1445-2197.2006.03649.x

[16] Hartgrink HH, Putter H, Klein Kranenbarg E, Bonenkamp JJ, van de Velde CJ. Value of palliative resection in gastric cancer. Br J Surg. 2002;89:1438–43. 12390389 10.1046/j.1365-2168.2002.02220.x

[17] Bonenkamp JJ, Sasako M, Hermans J, van de Velde CJ. Tumor load and surgical palliation in gastric cancer. Hepatogastroenterology. 2001;48:1219–21. 11677934

[18] Maekawa S, Saku M, Maehara Y, Sadanaga N, Ikejiri K, Anai H, et al. Surgical treatment for advanced gastric cancer. Hepatogastroenterology. 1996;43:178–86. 8682459

[19] Ouchi K, Sugawara T, Ono H, Fujiya T, Kamiyama Y, Kakugawa Y, et al. Therapeutic significance of palliative operations for gastric cancer for survival and quality of life. J Surg Oncol. 1998;69:41–4. 9762890 10.1002/(SICI)1096-9098(199809)69:1<41::AID-JSO8>3.0.CO;2-K

[20] Miner TJ, Jaques DP, Karpeh MS, Brennan MF. Defining palliative surgery in patients receiving noncurative resections for gastric cancer. J Am Coll Surg. 2004;198:1013–21. 15194084 10.1016/j.jamcollsurg.2004.02.007

[21] Moehler M, Galle PR, Gockel I, Junginger T, Schmidberger H. The multidisciplinary management of gastrointestinal cancer. Multimodal treatment of gastric cancer. Best Pract Res Clin Gastroenterol. 2007;21:965–81. 18070698 10.1016/j.bpg.2007.10.003

[22] Mahar AL, Coburn NG, Singh S, Law C, Helyer LK. A systematic review of surgery for non-curative gastric cancer. Gastric cancer: Official J Int Gastric Cancer Association Japanese Gastric Cancer Association. 2012;15(Suppl 1):S125–37. 10.1007/s10120-011-0088-3 22033891

[23] Sun J, Song Y, Wang Z, Chen X, Gao P, Xu Y, et al. Clinical significance of palliative gastrectomy on the survival of patients with incurable advanced gastric cancer: a systematic review and meta-analysis. BMC Cancer. 2013;13:577. 24304886 10.1186/1471-2407-13-577 PMC4235220

[24] Sobin L, Gospodarowicz M, Wittekind C. International Union Against Cancer (UICC) TNM classification of malignant tumours, 7th edition. 2010:117–26.

[25] Sobin L, Wittekind C. International Union Against Cancer (UICC) TNM classification of malignant tumours, 6th edition. 2002:99–106.

[26] Stang A. Critical evaluation of the Newcastle-Ottawa scale for the assessment of the quality of nonrandomized studies in meta-analyses. Eur J Epidemiol. 2010;25:603–5. 20652370 10.1007/s10654-010-9491-z

[27] Tierney JF, Stewart LA, Ghersi D, Burdett S, Sydes MR. Practical methods for incorporating summary time-to-event data into meta-analysis. Trials. 2007;8:16. 17555582 10.1186/1745-6215-8-16 PMC1920534

[28] Al-Amawi T, Swider-Al-Amawi M, Halczak M, Wojtasik P, Kładny J. Advisability of palliative resections in incurable advanced gastric cancer. Polski Przeglad Chirurgiczny. 2011;83:449–56. 22166719 10.2478/v10035-011-0070-0

[29] Cai SR, He YL, Huang MJ, Dong WG, Peng JS, Zhan WH et al. [Clinical values of palliative gastrectomy for late-staged gastric cancer]. Zhonghua Wai Ke Za Zhi [Chinese journal of surgery]. 2003; 41:27–9. 12760753

[30] Chang YR, Han DS, Kong SH, Lee HJ, Kim SH, Kim WH, et al. The value of palliative gastrectomy in gastric cancer with distant metastasis. Ann Surg Oncol. 2012;19:1231–9. 22045464 10.1245/s10434-011-2056-x

[31] Chen S, Li YF, Feng XY, Zhou ZW, Yuan XH, Chen YB. Significance of palliative gastrectomy for late-stage gastric cancer patients. J Surg Oncol. 2012;106:862–71. 22648960 10.1002/jso.23158

[32] Chen XJ, Chen GM, Wei YC, Yu H, Wang XC, Zhao ZK, et al. Palliative Gastrectomy versus Gastrojejunostomy for advanced gastric cancer with outlet obstruction: a propensity score matching analysis. BMC Cancer. 2021;21:188. 33622258 10.1186/s12885-021-07904-7 PMC7903659

[33] Chiu CF, Yang HR, Yang MD, Jeng LB, Yang TY, Sargeant AM et al. Palliative Gastrectomy Prolongs Survival of Metastatic Gastric Cancer Patients with Normal Preoperative CEA or CA19-9 Values: A Retrospective Cohort Study. Gastroenterology research and practice. 2016; 2016:6846027. 10.1155/2016/6846027 PMC5136406 27990157

[34] Hsu JT, Liao JA, Chuang HC, Chen TD, Chen TH, Kuo CJ, et al. Palliative gastrectomy is beneficial in selected cases of metastatic gastric cancer. BMC Palliat care. 2017;16:19. 28288593 10.1186/s12904-017-0192-1 PMC5348866

[35] Huang KH, Wu CW, Fang WL, Chen JH, Lo SS, Wang RF, et al. Palliative resection in noncurative gastric cancer patients. World J Surg. 2010;34:1015–21. 20145923 10.1007/s00268-010-0467-7

[36] Kamarajah SK, Markar SR, Phillips AW, Salti GI, Dahdaleh F, Griffiths EA. Palliative gastrectomy for metastatic gastric adenocarcinoma: a national population-based cohort study. Surgery. 2021;170:1702–10. 34389165 10.1016/j.surg.2021.07.016

[37] Kim DY, Joo JK, Park YK, Ryu SY, Kim YJ, Kim SK, et al. Is palliative resection necessary for gastric carcinoma patients? Langenbeck’s Archives Surg. 2008;393:31–5. 10.1007/s00423-007-0206-1 17593384

[38] Li Q, Zou J, Jia M, Li P, Zhang R, Han J, et al. Palliative gastrectomy and survival in patients with metastatic gastric Cancer: a propensity score-matched analysis of a large Population-based study. Clin Translational Gastroenterol. 2019;10:1–8. 10.14309/ctg.0000000000000048 PMC6602769 31116140

[39] Nie RC, Chen S, Yuan SQ, Chen XJ, Chen YM, Zhu BY, et al. Significant role of Palliative Gastrectomy in selective gastric Cancer patients with peritoneal dissemination: a propensity score matching analysis. Ann Surg Oncol. 2016;23:3956–63. 27380641 10.1245/s10434-016-5223-2

[40] Omori H, Tanizawa Y, Makuuchi R, Irino T, Bando E, Kawamura T, et al. Role of Palliative Resection in patients with Incurable Advanced Gastric Cancer who are unfit for Chemotherapy. World J Surg. 2019;43:571–9. 30298282 10.1007/s00268-018-4816-2

[41] Park JY, Yu B, Park KB, Kwon OK, Lee SS, Chung HY. Impact of Palliative Gastrectomy in patients with incurable gastric Cancer. Medicina (Kaunas, Lithuania). 2021; 57. 10.3390/medicina57030198 PMC7996496 33652574

[42] Peng W, Ma T, Xu H, Wu Z, Wu C, Sun G. Survival benefits of palliative gastrectomy in stage IV gastric cancer: a propensity score matched analysis. J Gastrointest Oncol. 2020;11:376–85. 32399278 10.21037/jgo.2020.01.07 PMC7212110

[43] Saidi RF, ReMine SG, Dudrick PS, Hanna NN. Is there a role for palliative gastrectomy in patients with stage IV gastric cancer? World J Surg. 2006;30:21–7. 16369718 10.1007/s00268-005-0129-3

[44] Yang K, Liu K, Zhang WH, Lu ZH, Chen XZ, Chen XL, et al. The value of Palliative Gastrectomy for gastric Cancer patients with Intraoperatively Proven Peritoneal Seeding. Medicine. 2015;94:e1051. 26166075 10.1097/MD.0000000000001051 PMC4504616

[45] Yang LP, Wang ZX, He MM, Jin Y, Ren C, Wang ZQ, et al. The survival benefit of palliative gastrectomy and/or metastasectomy in gastric cancer patients with synchronous metastasis: a population-based study using propensity score matching and coarsened exact matching. J Cancer. 2019;10:602–10. 30719157 10.7150/jca.28842 PMC6360412

[46] Yuan SQ, Nie RC, Chen S, Chen XJ, Chen YM, Xu LP, et al. Selective gastric Cancer patients with peritoneal Seeding Benefit from Gastrectomy after Palliative Chemotherapy: a propensity score matching analysis. J Cancer. 2017;8:2231–7. 28819425 10.7150/jca.18932 PMC5560140

[47] Wong BC, Lam SK, Wong WM, Chen JS, Zheng TT, Feng RE, et al. Helicobacter pylori eradication to prevent gastric cancer in a high-risk region of China: a randomized controlled trial. JAMA. 2004;291:187–94. 14722144 10.1001/jama.291.2.187

[48] Takahashi T, Saikawa Y, Kitagawa Y. Gastric cancer: current status of diagnosis and treatment. Cancers. 2013;5:48–63. 24216698 10.3390/cancers5010048 PMC3730304

[49] Sarela AI, Yelluri S. Gastric adenocarcinoma with distant metastasis: is gastrectomy necessary? Archives of surgery (Chicago, Ill: 1960). 2007; 142:143-9; discussion 9. 10.1001/archsurg.142.2.143 17309965

[50] Cowling J, Gorman B, Riaz A, Bundred JR, Kamarajah SK, Evans RPT, et al. Peri-operative outcomes and survival following palliative gastrectomy for gastric Cancer: a systematic review and Meta-analysis. J Gastrointest cancer. 2021;52:41–56. 32959118 10.1007/s12029-020-00519-4 PMC7900337

[51] Zheng C, Gao ZM, Huang HB, Li K, Liu XF. Prognostic significance of palliative gastrectomy in incurable advanced gastric cancer: a retrospective cohort study and meta-analysis. Eur Rev Med Pharmacol Sci. 2021;25:2299–312. 33755967 10.26355/eurrev_202103_25262

[52] Lasithiotakis K, Antoniou SA, Antoniou GA, Kaklamanos I, Zoras O. Gastrectomy for stage IV gastric cancer. A systematic review and meta-analysis. Anticancer Res. 2014;34:2079–85. 24778009

[53] Stillwell AP, Buettner PG, Ho YH. Meta-analysis of survival of patients with stage IV colorectal cancer managed with surgical resection versus chemotherapy alone. World J Surg. 2010;34:797–807. 20054541 10.1007/s00268-009-0366-y

